# Anemia, intractable vomiting, chronic diarrhea, and syndrome of inappropriate antidiuretic secretion: a diagnostic dilemma: Disseminated strongyloidosis in a patient with newly diagnosed HTLV infection—case report and review of literature

**DOI:** 10.1097/MD.0000000000009229

**Published:** 2017-12-29

**Authors:** Hassan Tariq, Muhammad Umar Kamal, Pavithra Reddy, Bharat Bajantri, Masooma Niazi, Ajsza Matela, Cosmina Zeana, Ariyo Ihimoyan, Anil Dev, Sridhar Chilimuri

**Affiliations:** a Department of Medicine; b Department of Pathology, Bronx Lebanon Hospital Center, Bronx, NY.

**Keywords:** disseminated strongyloidosis, human T-lymphotropic virus, hyperinfection syndrome, syndrome of inappropriate antidiuretic secretion

## Abstract

**Rationale:**

Strongyloidiasis hyperinfection and disseminated disease have high mortality rates due to several complications and early detection of Strongyloides infection is therefore prudent.

**Patient concerns:**

A 37-year-old male patient came with chronic diarrhea, intractable vomiting and was found to have hyponatremia, and anemia on the initial laboratory tests.

**Diagnoses:**

Further work up revealed syndrome of inappropriate antidiuretic secretion to be the cause of the hyponatremia in addition to gastrointestinal loses. His hospital course was complicated by persistent hyponatremia and later development of partial small bowel obstruction.

**Interventions:**

Considering his symptoms we had a suspicion of small bowel pathology for which he underwent an esophagogastroduodenoscopywith biopsies that revealed strongyloidosis as the cause of his symptoms. He was also found to have human T-cell lymphotropic virus infection, likely contributing to the disseminated disease.

**Outcomes:**

He was started on ivermectin with complete resolution of symptoms and improvement of hyponatremia.

**Lessons:**

It is very important to suspect Strongyloides infection in a patient presenting with syndrome ofinappropriate antidiuretic secretion as hyperinfection and disseminated disease can be life threatening without antihelmintic therapy.

## 1 Introduction

Strongyloidiasis is a common infection in the tropics and subtropics. The presentation of Strongyloides ranges from subclinical to severe and fatal disease in hyperinfection syndrome and disseminated strongyloidiasis, which have mortality rates of approximately 90%.^[1]^ If host is immunosuppressed, patients can develop disseminated disease or may lead to a “hyperinfection syndrome,” in which larval invasion of the peritoneum, liver, lungs, and central nervous system may occur, followed by bacterial peritonitis, meningitis, and septicaemia. Mortality rates are high, and early detection of Strongyloides infection is therefore prudent.^[2,3]^

## 2 Case presentation

A 37-year-old man presented to our hospital with abdominal pain for 2 weeks. The pain was periumbilical, nonradiating, 4/10 in intensity, intermittent with no aggravating or relieving factors. The abdominal pain was associated with nausea, vomiting, loss of appetite, and chronic diarrhea. He did not notice blood or mucous in his stool. The diarrhea was associated with a 50-pound weight loss over the past 6 months. Review of system was significant for persistent frontal headaches with on and off dizziness over the last few weeks. He did not have fever, neck stiffness, blurry vision, rash, trauma, or any other sick contacts.

Patient was recently admitted to another hospital with similar symptoms, and as per the records received, was treated for hypo-osmolar hyponatremia and discharged after the symptoms had improved. His past medical history was significant for hypertension. He had no previous surgical history. Family history was significant for hypertension in both parents. He drank 3 to 4 beers every weekend. He was born and raised in Honduras, came to the United States in 2004, and had recently moved from Connecticut to New York about 7 months ago. He had not traveled outside United States since he came to the United States and was living with his 2 sisters; none of whom had any similar symptoms.

On presentation the patient was afebrile, with heart rate of 89 beats/min, with blood pressure of 120/62 mm Hg, and an oxygen saturation of 98% on ambient air. On examination, he was cachectic, alert, and oriented, and had dry oral mucosa, sunken eyes, and appeared dehydrated. His cardiorespiratory examination was unremarkable. His abdomen was soft, mildly tender in the periumbilical area with hypoactive bowel sounds. Neurological examination was unremarkable. The initial labs on presentation are tabulated in Table 1.

**Table 1:**
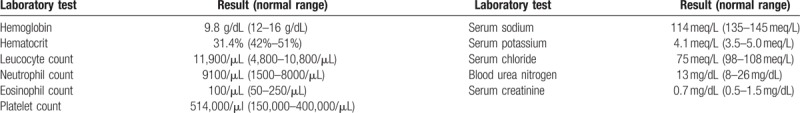
Initial laboratory values on presentation.

He was started initially on hypertonic saline for hyponateremia and possible neurological manifestations secondary to hypovolemic hyponateremia secondary to gastrointestinal losses and later switched to 0.9% normal saline once the serum sodium had improved with resolution of headache and dizziness. The patient was started on antiemetics for vomiting and antibiotics (metronidazole and ciprofloxacin) for diarrhea. Stool studies could not be collected due to patients’ noncooperation and later the diarrhea resolved after 2 days of hospitalization. He also had improvement in his vomiting.

**Figure 1:**
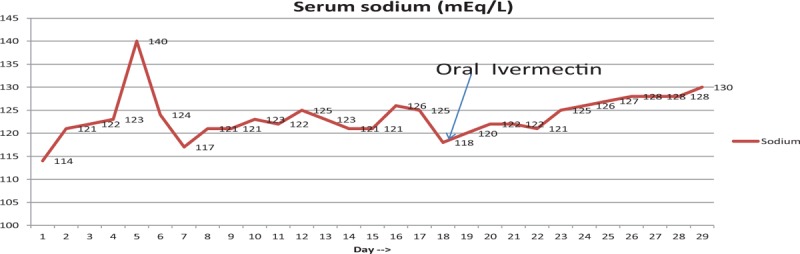
Serum sodium level during the hospitalization. Serum sodium value in mEq/L graphically represented over days. On day 18 patient was started on oral ivermectin with stable improvement in serum sodium value.

His serum sodium levels did not improve to normal values despite medical therapy (Fig. 1). To evaluate the cause of his persistent hyponatremia, further work up was done that revealed a serum osmolarity of 246 mOsm/kg (275–295 mOsm/kg), urine osmolality 177 mOsm/kg water (300–900 mOsm/kg water), urine sodium <20 meq/L (40–220 meq/L). He had subclinical hyperthyroidism (low TSH, normal T3, and free thyroxine levels). A cortisol stimulation test was done to rule out any adrenal insufficiency as a cause of hyponatremia, which showed appropriate response. The serum sodium levels during the hospitalization are shown in Figure 1.

**Figure 2:**
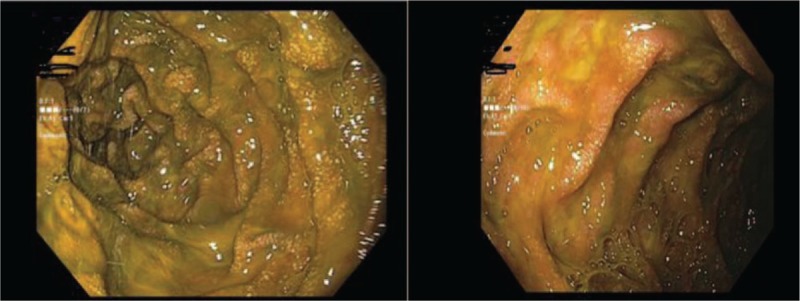
Edema and widespread whitish spots consistent with intestinal lymphangiectasia in the duodenum.

**Figure 3:**
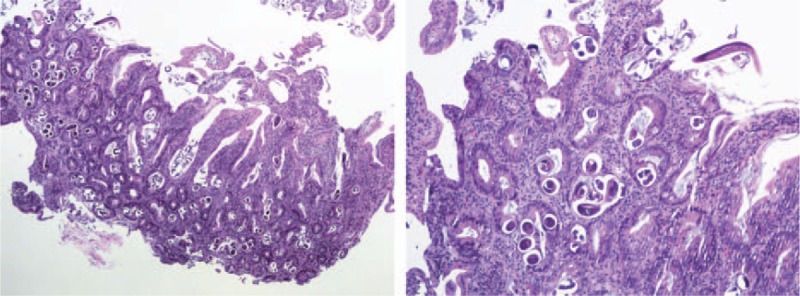
Strongyloides infection of the duodenum: Longitudinal (left) and transverse (right) cross sections of several worms and larvae lying within the intestinal lumen and crypts. The lamina propria shows neutrophils, eosinophils and mononuclear cells. [Hematoxylin and eosin stain low magnification 40× (left) and high magnification 100× (right)].

**Figure 4:**
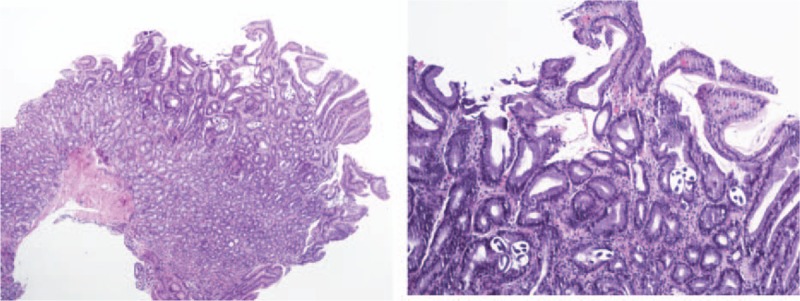
Gastric mucosa showing strongyloides larvae within the crypts. [Hematoxylin and eosin stain low magnification 40× (left) and high magnification 100× (right)].

The results were consistent with the presence of syndrome of inappropriate antidiuretic hormone secretion (SIADH) as the etiology of this patient's hyponateremia, in addition to the gastrointestinal losses; 0.9% normal saline was discontinued, and he was started on free water restriction. To evaluate the cause of SIADH, further work up including CT scan of head and chest to rule out potential causes of SIADH was performed which were unremarkable. The human immunodeficiency virus (HIV) and rapid plasma reagin (RPR) testing were also negative. His hospital course was complicated with sudden worsening of vomiting and diffuse abdominal pain. A CT scan of abdomen was done for further evaluation, which showed partial small bowel obstruction without any mass. A nasogastric tube was placed, and he was managed conservatively with resolution of the obstruction, but he persistently had intractable vomiting. Considering the history of chronic diarrhea, weight loss, intractable vomiting, he underwent an esophagogastroduodenoscopy (EGD) to evaluate for a possible small bowel pathology. Interestingly, we found edema and widespread whitish spots consistent with intestinal lymphangiectasia in the duodenum (Fig. 2). Biopsies were taken from the stomach and small bowel. He was started on restrictive medium chain triglyceride (MCT) diet (low fat, high protein diet). The pathology report from the GI biopsies was reported as presence of extensive strongyloidosis infection with lymphocytic and focal eosinophilic infiltrate (Figs. 3 and 4). He was started on ivermectin. It was recognized that he had systemic strongyloidosis with extensive gastrointestinal involvement as well as being the cause of his SIADH. We wanted to further evaluate the reason of systemic strongyloidosis as it is usually associated with immunosuppression. A human T-cell lymphotropic virus (HTLV) 1 and 2 serology was sent that was reported as positive. After starting treatment with ivermectin patient's symptoms improved with increase in serum sodium and patient was able to tolerate regular oral diet.

## 3 Discussion

*Strongyloides stercoralis* is commonly found in the tropical, subtropical, and warm temperate regions. According to an estimate, about 30 to 100 million people are infected worldwide.^[1]^ Higher percentage of patients infested with strongyloidiasis is observed in immigrants/refugees from tropical and subtropical countries (e.g., Southeast Asia, Africa, Middle East),^[2]^ and war veterans of World War II and the Vietnam War resided at or travelled to endemic areas.^[3]^ The prevalence of infected individuals ranges from 0% to 46.1% in immigrant populations in the United States as compared with the range of 0% to 6.1% among randomly selected US population.^[1,4,5]^ Our patient migrated to the United States about 12 years ago from South America, an endemic region for *S. stercoralis* infections.

*S. stercoralis* is a complex and unique nematode because it completes its entire life cycle within the human host. The invasive filariform larvae are found in soil, water, and feces; they penetrate the skin, and migrate to the lungs via venous circulation. They penetrate the alveoli, ascend through the tracheobronchial tree, and are swallowed. After reaching GI tract, the larvae mature into adult females, reside in the duodenal and jejunal mucosa, and lay eggs.^[6–8]^ Eggs hatch into rhabditiform larvae, which are either passed in stools or penetrate intestinal mucosa or the perineal skin area causing autoinfection.^[6,8]^ This mechanism of “autoinfection” is the probable cause of hyperinfection in our patient.

The host innate and adaptive immunity plays a central role in preventing hyperinfection and disseminated disease in strongyloidiasis. It stimulates Th-2 lymphocyte predominant immune response with production of cytokines, IgE antibodies, eosinophils, and mast cells, which execute expulsion, and killing of the parasite.^[9–11]^ Strongyloides antigens activate eosinophils via the innate immune response.^[12]^ After antigenic stimulation activated eosinophils enhance production of Th-2-specific cytokines including IL-4 and IL-5^[12,13]^. IL-4 induces class switching of B cells leading to production of IgE and IgG4 antibodies. Other cytokines like IL-8 attract neutrophils and contribute in killing of larvae.^[10–12]^ IgE promotes eosinophil migration,^[13]^ whereas IL-5 stimulates eosinophil growth and activation.^[12,13]^ Antibodies against Strongyloides, complement activation, and granulocytes via ADCC play an important role in protection against dissemination of infective larvae and development of hyperinfection. The delicate balance between the innate and adaptive immune system allows prolonged survival of the pathogen in the host gastrointestinal tract and prevents invasion.^[11,14]^ The dysregulation of the host immune system with loss of normal innate and adaptive immune response to worm infection predisposes patients to hyperinfection and dissemination syndromes.^[9,15,16]^ The immunologic deficiencies secondary to malnutrition, hypogammaglobinemia, diabetes, hematologic malignancies, use of immunosuppressive drugs, and HTLV-1 are associated with enhanced risk of hyperinfection and dissemination.^[14,17]^

The immunoglobulins also remarkably contribute to defense mechanisms against *S. stercoralis* larvae. In humans, lower levels IgM and IgG antibody levels were found in people with severe Strongyloides as compared with individuals with asymptomatic or mild symptomatic individuals.^[18]^

Similarly, it was seen that protective immunity in mice to the infective third larvae (L3) of *S. stercoralis* involved IgM.^[19]^ Approximately 50% of infected patients are without symptoms.^[6,8]^ The patients commonly experience gastrointestinal symptoms (anorexia, nausea, abdominal pain, flatulence, constipation, diarrhea, and weight loss). Advanced disease is responsible for causing malabsorption syndromes, paralytic ileus, intestinal obstruction, and gastrointestinal hemorrhage.^[6,8,20]^ Pulmonary symptoms (cough, dyspnea, wheezing, and hemoptysis) usually occur during the primary migration phase of larvae in the pulmonary parenchyma. The skin should be carefully examined in suspected cases for the urticarial/maculopapular or serpiginous rash known as Larva currens (racing larva).^[6,21,22]^ Our patient has predominantly GI symptoms along with SIADH. Activated Eosinophils play an essential role in protecting against *S. stercoralis* larvae^[23]^ by inducing the expression of major histocompatibility complex (MHC) class II and potentiating T cells for antigen-specific immune responses.^[24]^ Eosinophils act as APCs for the mediating the primary and secondary Th-2 immune responses against *S. stercoralis*^[24,25]^ and serve as an interface between innate and adaptive immune responses. It was found that eosinophil levels were lower in individuals with severe strongyloidiasis, as compared with those with asymptomatic individuals.^[26]^ In another study, it was seen that approximately 75% of patients with chronic strongyloidiasis have peripheral eosinophilia or elevated total IgE levels.^[12,15]^ Therefore, it is assumed that eosinophil levels may play definitive role in preventing and combating *S. stercoralis* infection. In our patient, the eosinophil levels were in the lower range initially but increased subsequently after starting antihelmintic activity.

Hyperinfection syndrome is defined as accelerated autoinfection, whereas disseminated disease refers to the massive migration of infective larvae outside of the usual route after invading the gut wall to various organs, including the lungs and central nervous system (CNS).^[6]^ Hematogenous dissemination of enteric bacteria through damaged intestinal mucosa or the invasive larvae itself can facilitate translocation of enteric bacteria^[21]^ and are associated with high mortality rate (up to 87%) due to secondary to bacteremia/sepsis or meningitis caused by enteric pathogens.^[27,28]^

Development of disseminated strongyloidiasis is usually associated with immunosuppressed state, such as malnutrition; hypogammaglobulinemia; diabetes, chronic alcoholism, renal failure, advanced age, drug therapy with corticosteroids or anticancer medications; hematologic malignancies; solid organ or bone marrow transplantation; human T-lymphotropic virus-1 infection or HIV infection.^[8,29]^ Our patient had history of alcohol abuse and HTLV-1 infection, which might have had contributed to disseminated disease.

The infection of HTLV-1 in patients with Strongyloides creates a unique imbalance of the immune responses resulting in increased susceptibility of host to disseminated disease.^[11,30,31]^ It causes increased interferon-gamma (IFN-γ) production while decreasing levels of interleukin-4 (IL-4), IL-5, and IgE antibodies.^[32–34]^ HTLV-1 can cause immunologic switching from Th-2 responses to Th-1 responses which favors hyperinfection. The suppressed Th-2 responses result in decreased serum concentrations of IL-4, IL-5, IL-13, and IgE antibodies against *S. stercoralis*.^[34–37]^ The reduced levels of IL-4 and IgE progressively diminish mast cell function, eosinophil recruitment, and the efficacy of the host immune system to kill the parasite.^[11,38]^ The activated Th-1 responses increase the expression of IFN-γ and tumor growth factor (TGF-β), decrease the serum levels of IgE antibodies against *S. stercoralis* and IgG4, impair the immune response against strongyloides, and alter therapeutic responses.^[39]^ Our patient was infected with HLTV-1, which might be the primary reason of hyperinfection.

There are no vivid mechanisms describing the development of tumors in strongyloidosis patients, but some malignancies have been reported with hyperinfection syndrome. It includes primary organ cancers such as lung and gastrointestinal and primary hematologic malignancies before starting the immunosuppressive chemotherapy or steroids.^[9,15,40]^ Although cases of human immunodeficiency virus (HIV) and *S. stercoralis* have been reported,^[11,41–43]^ the immunoregulatory mechanisms in HIV patients leading to disseminated strongyloidiasis remain controversial. It is assumed that immunocompromised HIV individuals are at high risk of developing hyperinfection with *S. stercoralis*, but this risk seems less than expected. A study showed significant negative rank correlation between CD4+ cell counts and the percentage of free living worms and the absence of disseminated strongyloidiasis in advanced HIV infection.^[44]^ Another study in Uganda observed that *S. stercoralis* was not associated with higher viral load.^[45]^

This might be because HIV infects CD4 T lymphocytes and induces T-cell destruction^[12]^ which results in sudden decrease in Th-1 lymphocytes as compared with Th-2 lymphocytes. The Th-1-mediated cytokines production is markedly affected than Th-2-mediated cytokine release which promotes mucosal regulation of strongyloidiasis.^[13]^ In addition, HIV infection causes elevated levels of IgE, eosinophils, and in turn suppresses the development of Strongyloides larvae in the gut necessary for autoinfection.^[13,14]^ In contrast, cases of hyperinfection have been reported in HIV patients^[46]^ and advances HIV illnesses.^[47]^

To the best of our knowledge, only 8 cases^[48–55]^ of the syndrome of inappropriate secretion of antidiuretic hormone (SIADH) and Strongyloides infection have been reported in the literature (Table 2). However, there is extensive involvement of the lung parenchyma or central nervous system seen in most of these cases. In our patient and the case described by Khanna et al, there was no involvement of CNS and lungs, therefore mechanism through which *S. stercoralis* caused SIADH is not clear. In the present case, the SIADH developed following chronic infection with *S. stercoralis* accompanied by anorexia, malnutrition, emaciation, and constipation with partial bowel obstruction. These symptoms and hyponatremia improved after treatment for *S. stercoralis*.

**Table 2:**
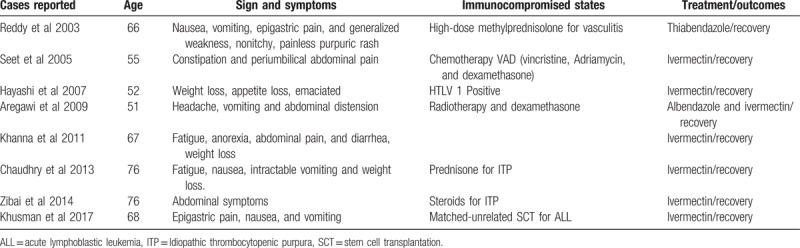
Previous reported cases of SIADH in Strongyloides infection.

Diagnosis of *S. stercoralis* infection can be challenging as single fecal sample examination has low sensitivity (75.9%) because of intermittent parasite burden and excretion.^[7,8,56]^ Therefore, if there is high suspicion of infection, up to 3 stool samples or multiple duodenal fluid aspirates increase the sensitivity up to 92%.^[39,56]^ Stool culture on a blood agar plate is a very sensitive technique because it identifies the motility of Strongyloides larvae to diagnose infection.^[39]^ The diagnostic sensitivity is enhanced using modern techniques of polymerase chain reaction (PCR) and enzyme-linked immunosorbent assay (ELISA)-based immunoassay.^[56–58]^ Eosinophilia is nonspecific which can be seen in approximately 75% of patients with uncomplicated Strongyloidiasis infection and can be absent in immunocompromised individuals, especially those treated with steroids.^[59,60]^ Cases of disseminated strongyloidiasis necessitate further evaluation of all organs which might be infected; this includes BAL, duodenal/Jenjunal fluid sampling, and/or biopsies or skin biopsy.^[56]^ The diagnoses of strongyloidiasis in our case were made with esophagogastroduodenoscopy and biopsy of gastric and duodenal mucosa which showed many *S. stercoralis* larvae.

Ivermectin is more effective in eradicating infection; including patients who did not respond well to thiabendazole therapy. Ivermectin has shown better response when given 200 μg/kg/d orally for 1 to 2 days for complicated intestinal strongyloidiasis or 200 μg/kg/d orally for 2 weeks for hyperinfection or disseminated infections until stool and/or sputum examinations are negative (CDC). One study demonstrated a higher (83%) cure rate with 200 μg/kg as a single-dose ivermectin as compared with 400 mg/d for 3 days albendazole (38%).^[61]^ Our patient achieved cure after 14 doses of ivermectin verified by a stool testing along with improvement of vomiting and serum sodium levels. Patients who experience frequent relapses may benefit from monthly treatment regimens.^[8,62]^ Cases of disseminated strongyloidiasis not responding to conventional regimens might benefit from the combination of ivermectin and albendazole and should be continued until there is evidence of parasite eradication.^[46,47,63]^ It is proposed that patient should be followed with serial antibody testing after curative treatment. But further research is needed to establish this strategy as a marker of eradication.^[64,65]^

## 4 Conclusions

SIADH has been described with systemic strongyloidiasis hyperinfection and likely etiology attributed to CNS or pulmonary involvement.^[48,49,51,66]^ Our case had only GI involvement without CNS or pulmonary features, therefore mechanism through which Strongyloides caused SIADH remains unknown. Additional studies are needed to unveil the mechanisms of SIADH and human strongyloidiasis. It is very important to suspect strongyloides infection in a patient presenting with SIADH as hyperinfection and disseminated disease can be life threatening without antihelmintic therapy.

## References

[1] http://www.cdc.gov/parasites/strongyloides/health_professionals/2016.

[2] Dawson-Hahn EE Greenberg SL Domachowske JB Eosinophilia and the seroprevalence of schistosomiasis and strongyloidiasis in newly arrived pediatric refugees: an examination of Centers for Disease Control and Prevention screening guidelines. J Pediatr 2010;156:1016–8. 20400098 10.1016/j.jpeds.2010.02.043

[3] Gill GV Welch E Bailey JW Chronic Strongyloides stercoralis infection in former British Far East prisoners of war. QJM 2004;97:789–95. 15569810 10.1093/qjmed/hch133

[4] Liu LX Weller PF Strongyloidiasis and other intestinal nematode infections. Infect Dis Clin North Am 1993;7:655–82. 8254165

[5] Nuesch R Zimmerli L Stockli R Imported strongyloidosis: a longitudinal analysis of 31 cases. J Travel Med 2005;12:80–4. 15996452 10.2310/7060.2005.12204

[6] Segarra-Newnham M Manifestations, diagnosis, and treatment of Strongyloides stercoralis infection. Ann Pharmacother 2007;41:1992–2001. 17940124 10.1345/aph.1K302

[7] Siddiqui AA Berk SL Diagnosis of Strongyloides stercoralis infection. Clin Infect Dis 2001;33:1040–7. 11528578 10.1086/322707

[8] Concha R Harrington W Jr Rogers AI Intestinal strongyloidiasis: recognition, management, and determinants of outcome. J Clin Gastroenterol 2005;39:203–11. 15718861 10.1097/01.mcg.0000152779.68900.33

[9] Montes M Sawhney C Barros N Strongyloides stercoralis: there but not seen. Curr Opin Infect Dis 2010;23:500–4. 20733481 10.1097/QCO.0b013e32833df718 PMC2948977

[10] Porto AF Neva FA Bittencourt H HTLV-1 decreases Th2 type of immune response in patients with strongyloidiasis. Parasite Immunol 2001;23:503–7. 11589779 10.1046/j.1365-3024.2001.00407.x

[11] Iriemenam NC Sanyaolu AO Oyibo WA Strongyloides stercoralis and the immune response. Parasitol Int 2010;59:9–14. 19892034 10.1016/j.parint.2009.10.009

[12] Montes M Sanchez C Verdonck K Regulatory T cell expansion in HTLV-1 and strongyloidiasis co- infection is associated with reduced IL-5 responses to Strongyloides stercoralis antigen. PLoS Negl Trop Dis 2009;3:e456. 19513105 10.1371/journal.pntd.0000456 PMC2686100

[13] Siegel MO Simon GL Is human immunodeficiency virus infection a risk factor for Strongyloides stercoralis hyperinfection and dissemination. PLoS Negl Trop Dis 2012;6:e1581. 22860137 10.1371/journal.pntd.0001581 PMC3409107

[14] Weatherhead JE Mejia R Immune Response to Infection with Strongyloides stercoralis in Patients with Infection and Hyperinfection. Curr Trop Med Rep 2014;1:229–33.

[15] Ramanathan R Nutman T Strongyloides stercoralis infection in the immunocompromised host. Curr Infect Dis Rep 2008;10:105–10. 18462583 10.1007/s11908-008-0019-6 PMC3401551

[16] Nabha L Krishnan S Ramanathan R Prevalence of Strongyloides stercoralis in an urban US AIDS cohort. Pathog Glob Health 2012;106:238–44. 23265425 10.1179/2047773212Y.0000000031 PMC4001591

[17] Le M Ravin K Hasan A Single donor-derived strongyloidiasis in three solid organ transplant recipients: case series and review of the literature. Am J Transplant 2014;14:1199–206. 24612907 10.1111/ajt.12670 PMC10167799

[18] Carvalho EM Andrade TM Andrade JA Immunological features in different clinical forms of strongyloidiasis. Trans R Soc Trop Med Hyg 1983;77:346–9. 6623593 10.1016/0035-9203(83)90162-1

[19] Herbert DR Nolan TJ Schad GA The role of B cells in immunity against larval Strongyloides stercoralis in mice. Parasite Immunol 2002;24:95–101. 11874564 10.1046/j.0141-9838.2001.00441.x

[20] Mahmoud AA Strongyloidiasis. Clin Infect Dis 1996;23:949–52. 8922784 10.1093/clinids/23.5.949

[21] Galimberti R Ponton A Zaputovich FA Disseminated strongyloidiasis in immunocompromised patients—report of three cases. Int J Dermatol 2009;48:975–8. 19702983 10.1111/j.1365-4632.2009.04082.x

[22] Ganesh S Cruz RJ Jr Strongyloidiasis: a multifaceted disease. Gastroenterol Hepatol (N Y) 2011;7:194–6. 21528049 PMC3079152

[23] Satoh M Toma H Kiyuna S Association of a sex-related difference of Strongyloides stercoralis-specific IgG4 antibody titer with the efficacy of treatment of strongyloidiasis. Am J Trop Med Hyg 2004;71:107–11. 15238698

[24] Padigel UM Lee JJ Nolan TJ Eosinophils can function as antigen-presenting cells to induce primary and secondary immune responses to Strongyloides stercoralis. Infect Immun 2006;74:3232–8. 16714550 10.1128/IAI.02067-05 PMC1479274

[25] Padigel UM Hess JA Lee JJ Eosinophils act as antigen-presenting cells to induce immunity to Strongyloides stercoralis in mice. J Infect Dis 2007;196:1844–51. 18190266 10.1086/522968 PMC3154724

[26] Shi H-Z Eosinophils function as antigen-presenting cells. J Leukoc Biol 2004;76:520–7. 15218055 10.1189/jlb.0404228

[27] Somin M Neogolani V Zimhony O Fatal recurrent bacterial meningitis: a complication of chronic Strongyloides infection. Eur J Intern Med 2008;19:e42–3. 18848169 10.1016/j.ejim.2007.07.009

[28] Link K Orenstein R Bacterial complications of strongyloidiasis: Streptococcus bovis meningitis. South Med J 1999;92:728–31. 10414486 10.1097/00007611-199907000-00016

[29] Yoshida H Endo H Tanaka S Recurrent paralytic ileus associated with strongyloidiasis in a patient with systemic lupus erythematosus. Mod Rheumatol 2006;16:44–7. 16622724 10.1007/s10165-005-0447-1

[30] Nakada K Kohakura M Komoda H High incidence of HTLV antibody in carriers of Strongyloides stercoralis. Lancet 1984;1:633. 10.1016/s0140-6736(84)91030-4 6142338

[31] Hirata T Uchima N Kishimoto K Impairment of host immune response against strongyloides stercoralis by human T cell lymphotropic virus type 1 infection. Am J Trop Med Hyg 2006;74:246–9. 16474078

[32] Neva FA Oliveira Filho J Gam AA Interferon-γ and interleukin-4 responses in relation to serum IgE levels in persons infected with human T lymphotropic virus type I and Strongyloides stercoralis. J Infect Dis 1998;178:1856–9. 9815251 10.1086/314507

[33] Evering T Weiss L The immunology of parasite infections in immunocompromised hosts. Parasite Immunol 2006;28:549–65. 17042927 10.1111/j.1365-3024.2006.00886.x PMC3109637

[34] Carvalho Filho EMd, Porto MAdF. Epidemiological and clinical interaction between HTLV-1 and Strongyloides stercoralis. 2004. 10.1111/j.0141-9838.2004.00726.x 15771684

[35] Loutfy MR Wilson M Keystone JS Serology and eosinophil count in the diagnosis and management of strongyloidiasis in a non-endemic area. Am J Trop Med Hyg 2002;66:749–52. 12224585 10.4269/ajtmh.2002.66.749

[36] Wagenvoort J Houben H Boonstra G Pulmonary superinfection withStrongyloides stercoralis in an immunocompromised retired coal miner. Eur J Clin Microbiol Infect Dis 1994;13:518–9. 7957278 10.1007/BF01974648

[37] Nolan TJ Bhopale VM Rotman HL Strongyloides stercoralis: high worm population density leads to autoinfection in the jird (Meriones unguiculatus). Exp Parasitol 2002;100:173–8. 12173402 10.1016/s0014-4894(02)00014-0

[38] Rivero F Kremer L Allende L [Strongyloides stercoralis and HIV: a case report of an indigenous disseminated infection from non-endemic area]. Rev Argent Microbiol 2005;38:137–9. 17152211

[39] Sato Y Kobayashi J Toma H Efficacy of stool examination for detection of Strongyloides infection. Am J Trop Med Hyg 1995;53:248–50. 7573706 10.4269/ajtmh.1995.53.248

[40] Keiser PB Nutman TB Strongyloides stercoralis in the Immunocompromised Population. Clin Microbiol Rev 2004;17:208–17. 14726461 10.1128/CMR.17.1.208-217.2004 PMC321465

[41] Sarangarajan R Ranganathan A Belmonte AH Strongyloides stercoralis infection in AIDS. AIDS Patient Care STDS 1997;11:407–14. 11361861 10.1089/apc.1997.11.407

[42] Ferreira MS Nishioka Sde A Borges AS Strongyloidiasis and infection due to human immunodeficiency virus: 25 cases at a Brazilian teaching hospital, including seven cases of hyperinfection syndrome. Clin Infect Dis 1999;28:154–5. 10028097 10.1086/517188

[43] Ohnishi K Kogure H Kaneko S Strongyloidiasis in a patient with acquired immunodeficiency syndrome. J Infect Chemother 2004;10:178–80. 15290458 10.1007/s10156-004-0312-8

[44] Viney ME Brown M Omoding NE Why does HIV infection not lead to disseminated strongyloidiasis? J Infect Dis 2004;190:2175–80. 15551217 10.1086/425935

[45] Brown M Kizza M Watera C Helminth infection is not associated with faster progression of HIV disease in coinfected adults in Uganda. J Infect Dis 2004;190:1869–79. 15499545 10.1086/425042

[46] Hughes R McGuire G Delayed diagnosis of disseminated strongyloidiasis. Intensive Care Med 2001;27:310–2. 11280656 10.1007/s001340000798

[47] Pornsuriyasak P Niticharoenpong K Sakapibunnan A Disseminated strongyloidiasis successfully treated with extended duration ivermectin combined with albendazole: a case report of intractable strongyloidiasis. Southeast Asian J Trop Med Public Health 2004;35:531–4. 15689061

[48] Reddy TS Myers JW Syndrome of inappropriate secretion of antidiuretic hormone and nonpalpable purpura in a woman with Strongyloides stercoralis hyperinfection. Am J Med Sci 2003;325:288–91. 12792249 10.1097/00000441-200305000-00007

[49] Seet RC Gong LL Tambyath PA Image of the month. Strongyloides stercoralis hyperinfection and syndrome of inappropriate secretion of antidiuretic hormone. Gastroenterology 2005;128:252. 10.1053/j.gastro.2004.11.034 15633117

[50] Hayashi E Ohta N Yamamoto H Syndrome of inappropriate secretion of antidiuretic hormone associated with strongyloidiasis. Southeast Asian J Trop Med Public Health 2007;239–46. 17539272

[51] Aregawi D Lopez D Wick M Disseminated strongyloidiasis complicating glioblastoma therapy: a case report. J Neurooncol 2009;94:439–43. 19333553 10.1007/s11060-009-9878-4

[52] Khanna S Sedlack RE Mangan TF An unusual case of SIADH. Gastroenterol Hepatol (N Y) 2011;7:191–3. 21528048 PMC3079151

[53] Chowdhury DN Dhadham GC Shah A Syndrome of inappropriate antidiuretic hormone secretion (SIADH) in Strongyloides stercoralis hyperinfection. J Glob Infect Dis 2014;6:23–7. 24741227 10.4103/0974-777X.127945 PMC3982351

[54] Zibaei M Whats new in global infectious diseases? Strongyloidiasis and syndrome of inappropriate antidiuretic hormone secretion (SIADH). J Glob Infect Dis 2014;6:1–2. 24741222 10.4103/0974-777X.127940 PMC3982347

[55] Khushman M Morris MI Diaz L Syndrome of inappropriate anti-diuretic hormone secretion secondary to strongyloides stercoralis infection in an allogeneic stem cell transplant patient: a case report and literature review. Transplant Proc 2017;49:373–7. 28219601 10.1016/j.transproceed.2016.12.012

[56] Krolewiecki AJ Ramanathan R Fink V Improved diagnosis of Strongyloides stercoralis using recombinant antigen-based serologies in a community-wide study in northern Argentina. Clin Vaccine Immunol 2010;17:1624–30. 20739501 10.1128/CVI.00259-10 PMC2952987

[57] Sato Y Takara M Otsuru M Detection of antibodies in strongyloidiasis by enzyme-linked immunosorbent assay (ELISA). Trans R Soc Trop Med Hyg 1985;79:51–5. 3992642 10.1016/0035-9203(85)90233-0

[58] Genta RM Predictive value of an enzyme-linked immunosorbent assay (ELISA) for the serodiagnosis of strongyloidiasis. Am J Clin Pathol 1988;89:391–4. 3348175 10.1093/ajcp/89.3.391

[59] Hallsworth MP Litchfield TM Lee TH Glucocorticoids inhibit granulocyte-macrophage colony-stimulating factor-1 and interleukin-5 enhanced in vitro survival of human eosinophils. Immunology 1992;75:382–5. 1551701 PMC1384724

[60] Wallen N Kita H Weiler D Glucocorticoids inhibit cytokine-mediated eosinophil survival. J Immunol 1991;147:3490–5. 1940348

[61] Datry A Hilmarsdottir I Mayorga-Sagastume R Treatment of Strongyloides stercoralis infection with ivermectin compared with albendazole: results of an open study of 60 cases. Trans R Soc Trop Med Hyg 1994;88:344–5. 7974685 10.1016/0035-9203(94)90110-4

[62] Shimasaki T Chung H Shiiki S Five cases of recurrent meningitis associated with chronic strongyloidiasis. Am J Trop Med Hyg 2015;92:601–4. 25548379 10.4269/ajtmh.14-0564 PMC4350558

[63] Lim S Katz K Krajden S Complicated and fatal Strongyloides infection in Canadians: risk factors, diagnosis and management. CMAJ 2004;171:479–84. 15337730 10.1503/cmaj.1031698 PMC514646

[64] Biggs BA Caruana S Mihrshahi S Management of chronic strongyloidiasis in immigrants and refugees: is serologic testing useful? Am J Trop Med Hyg 2009;80:788–91. 19407125

[65] Karunajeewa H Kelly H Leslie D Parasite-specific IgG response and peripheral blood eosinophil count following albendazole treatment for presumed chronic strongyloidiasis. J Travel Med 2006;13:84–91. 16553594 10.1111/j.1708-8305.2006.00004.x

[66] Vandebosch S Mana F Goossens A Strongyloides Stercoralis infection associated with repititive bacterial meningitis and SIADH: a case report. Acta Gastroenterol Belg 2008;71:413–7. 19317285

